# 

**Published:** 1902-04-05

**Authors:** 


### The Hospital

A JOURNAL OF
THE MEDICAL SCIENCES AND HOSPITAL ADMINISTRATION,

INCLUDING

A SPECIAL SECTION FOR NURSES.

IN TWO VOLUMES ANNUALLY.

EDITED BY

SIR HENRY BURDETT, K.C.B.,

AND

SOLOMON C. SMITH, M.D.

LIBRARY
SURGEON GENERALS OFFICE
JAN. 30. 1903
182006.

VOLUME XXXII.

APRIL TO SEPTEMBER 1902.

London:
PUBLISHED BY THE SCIENTIFIC PRESS (LIMITED), AT THE HOSPITAL BUILDING,
28 & 29 SOUTHAMPTON STREET, STRAND, W.C.

MDCCCCII.

W1 HO69X

### INDEX TO VOL. XXXII., 1902.

*** Articles under the general heading *Hospital Clinics and Medical Progress* are distinguished by the initial (C.) for *Hospital Clinics*, and by (P.) for the rest of the series. (W.) distinguishes the *Institutional Workshop* articles, and (A.) the *Annotations.*

Abdomen, Gunshot Wounds of the (P.), 187
Abdominal Affections, The Quiescent Period in Acute (C.), 96
Abiotrophy (C.), 61, 378
Abortion, Tubal, Clinical Lectures on (C.), 271
Abscess, Stitch, Prevention of (P.), 186
Addenbrooke's Hospital, Cambridge (W.), 141
Addison's Anæmia (C.), 149
Adenoids, The Removal of (A.), 221
Advantages of a Broken Head (A.), 163
Advertising in Various Grades, 268
Afforestation of Water-bearing Areas (A.), 35
Alcohol, Tuberculosis, and the Public House (C.), 429
Alcoholism and its Cures (A.), 235
Alexandra Hospital for Children (W.), 158
Aliens and the State (A.), 217
Alopecia Areata (P.), 311, 452
Amaurosis, Alcoholic (P.), 101
American Pay Patients (A.) 217
Amputation in the Course of Diabetes (C.), 23
Amputation at the Hip Joint (C.) 239
Amputation, Complete, of Two Limbs (C.), 221
Anæsthetics, Progress in (P.), 274
Anæsthetics in Short Operations (C.), 430
Anal Fissure (P.), 451
Anastomosis, Intestinal (P.), 241
Aneurysm, Arterio-venous (C.), 114
Ankle-clonus (P.), 433
Anti-toxin at Colchester, 18
Anti-typhoid Inoculation (C.), 415
Anti-vivisection Methods (W.), 244, 264
Antrum, Chronic Suppuration of the (C.), 62
Appendicitis (C.), 237, (P.), 240
Appendicitis, American Views on (C.), 238
Appendicitis, Convalesence after (C.), 288
Appendicitis, The Diagnosis of (C.), 255
Appendicitis, Leucocytosis in (C.), 289
Appendicitis, in Connection with Pelvic Disease (P.), 419
Appendix, A new Use for the, (C.), 376
Appendix, Vermiform, Surgery of the (P.), 240
Army Medical College for London, An (A.), 111
Army Medical Service, The (A.), 391
Army Medical Service, The Reorganisation of the (A.), 3
Around the Hospitals (W.), 124, 230, 245, 263, 317, 350
Arsenic and Cancer (C.), 289
Arsenic in the Human Body (C.), 186
Arthritis Accompanying Ophthalmia Neonatorum (C), 167
Arthritis, Acute Septic, of the Knee (C.), 272
Arthritis, Rheumatoid (P.), 98
Asthenopia (P.), 292
Atropine Drops, Death from the Use of (C.), 221
Awkward Problem, An (A.), 19
Bacteria in the Breath (P.), 98
Bacteria in Food Stuffs (P.), 418
Bacteriology, Progress in (P.), 84, 98, 188, 208, 275, 418
Banti's Disease (C), 394
Bedstead, A New Mechanical (W.), 369
Beer and Cancer (C.), 114
Benedikt's Syndrome (P.), 398
Beriberi (C.), 448
Bilharzia Disease (C.), 115
Birmingham Guardians and Labour Colonies (A.), 355
Birth Rates in Different Classes of Society (A.), 445
Bladder and Urethra, Treatment of Rupture of the (P.), 116
Blood Counting in General Practice (C.), 149
Boiled Water for Troops (A.), 93
Bonesetters and their Methods (C.), 254
Book World (*Books, Magazines, and Pamphlets Reviewed*)
Abbot. Tommy Cornstalk, 102
Barbour. *See Knopf.*
Bell-Ranske. Health, Speech, and Song, 454
Bennet. Massage in Recent Fractures, 383
Brooks. Elements of Mind, 331
Brown. The Case for Vaccination, 399
Burdett's Hospitals and Charities, 50
Campbell. Intemperance, 28
Carwardine. *See Pye.*
Cheadle (revised by Poynton). Artificial Feeding of Infants, 172
Choksy. Report on Leprosy, 102
Collinson. Facts about Flogging, 382
Colton. The Human Body, 226
Dorland. American Illustrated Dictionary of the Terms Used in Medicine, etc., 399
Fagge. The Pocket Gray or Anatomists' Vade Mecum, 190
Fox. Photographic Atlas of Diseases of the Skin, 172
French. Desmonde, M.D., 190
Galen (Lucas). Hospital Sketches, 120
Gibson. The Nordrach Treatment, 312
Gray. *See Fox.*
Greenwood. The Prevention of Infection in Public Vehicles, 330
Gümpel. Small-pox, 454
Hayward. Protoplasm, 436
Hichens. Some Results of the Nordrach Treatment in Ireland, 382
How. A Hero of Donegal: Memoir of Dr. William Smyth 226
Hutchinson and Rainy. Clinical Methods, 190
Keetley. Orthopædic Surgery, 274
Keith. Human Embryology and Morphology, 364
Knopf (translated by Barbour). Tuberculosis as a Disease of the Masses, 226
Lehfeldt. Text Book of Physics, 294
Matriculation Directory, 1902, 364
May. Official Guide to Llandudno, 102
Medical Annual, 1902, 50
Mercier. Text Book of Insanity, 260
Mortimer. Tendencies to Consumption, 364
Murray. Quain's Dictionary of Medicine, 28
Muthu. Some Results of the Sanatorium Treatment of Phthisis, 364
Newsholme. Hygiene and Public Health, 454
Paget. Selected Essays and Addresses. 190
Peters. Manipulation (or Massage) 120
Poore. The Earth in Relation to Contagia, 382
Poynton. *See Cheadle*
Pye (revised by Carwardine). Elementary Bandaging and Surgical Dressing, 154
Quain. *See Murray*
Rainy. *See Hutchinson.*
Rotch. Pediatrics, 120
Savill. Clinical Lectures on Neurasthenia, 437
Small-pox, 294
Small pox Insurance Diary, 102
Smith. Scientific Research, 172
Thompson. Poison Romance and Poison Mysteries, 260
Todd. The Way of Escape, 312
Turner. Manual of Practical Medical Electricity, 330
Vinrace. The War against Consumption, 154
Walker. Introduction to Dermatology 399
Walsh. The Hair and its Diseases, 436
Whiteway. Recent Object Lessons in Penal Science, 154
Year Book of Scientific and Learned Societies 312
Bracing or "Relaxing" (A.), 373
Brain and Nerves, Progress in Surgery of the (P.) 452
Breast Cancer, Operation in (C.), 131
Bright's Disease, Eruptions in (P.), 451
Bright's Disease and Surgical Treatment (P.), 258
British Home and Hospital for Incurables (W.), 140
British Lying-in Hospital (W.), 31
British Medical Association, The (A.), 201; (C.), 305: 319; (C.), 323
Brompton Consumption Hospital (W.), 177
Bronchiectasis, The Treatment of (C.), 61
Broncho-Pneumonia, Acute Infantile (C.), 447
Bronchoscopy (C), 323
Bunion (C), 43
Bunions, The Radical Cure of (C.), 113
Calculus, Encysted Vesical (P.), 100
Calculus, Renal (P.), 99
Cancer (P.) 361
Cancer, An Experiment in Regard to (C), 274
Cancer, Gastric (P), 11
Cancer, Inoperable, Treatment of (C.), 325
Cancer Investigation, The (A.), 35
Cancer, Origin of (C.), 37
Cancer, Problems in (C.), 239
Cancer, Progress in (P.), 64, 242, 256, 415
Cancer Research, 91, 301
Cancer of the Stomach (P.), 170
Cancer of the Uterus (C.), 219, 253, 342
Carcinoma (P.), 379
Carcinoma, The Vital Theory of the Causation of (C.). 376
Cardiac Failure in Diphtheria (C.), 95
Cartilage, Loose, in the Knee Joint, Treatment of (C.), 46
Cataract, Extraction of, in India (P.), 100
Cerebellar Ataxy; Hereditary (P.), 152
Cerebral Cortex (P.) 117
Cerebral Tumours (P.), 453
Charing Cross Hospital (W.), 245
Chelsea Hospital for Women (W.), 177
Chemical Physiology (C.), 449
Chemists' Exhibition, The (W.), 213
Child-Hawkers, 437
Chloroform Administration, 58
Cholera in Egypt, 302
Cholera at Mecca (A.), 3
Chorea (P.), 99
Chronic Student, The (A.), 163
Chronometry of Life, The, 372
Cirrhosis (P.), 46
Civilisation, Our Sham (A.), 201, *and see p.* 247
Club Foot, The Treatment of (C.), 220
Coley's Fluid in Sarcoma (C.), 149
Compulsory Notification of Consumption (A.), 355
Compulsory but Unpaid Professional Services, 426
"Conservative" Obstetrics (C.), 62
Contract Practice, 200
Corns (P.), 311
Coronation and the Weather, The, 199
Cotton-wool Sandwiches (C.), 8
Coxa Vara (C.), 375
Cream of the Workers, The (A.), 445
Cryoscopy in Genito-Urinary and Abdominal Surgery (P.), 99
Cures for Inebriates (A.), 303
Cystitis (P.), 275
Danger to South Africa, Another (A.), 391
Deaf Children (A.), 59
Deafness Cured by Cessation of Smoking (P.), 187
Decadence of the "Civilised" Negro (C.), 430
Deciduoma Malignum (P.), 432
Degrees and Diplomas, 400
Dermatitis (P.), 310, 311
Dermatitis caused by Plants (C.), 343
Dermatoses in Special Diseases (P.), 451
Diabetes, Eruptions in (P.), 452
Diabetes, Progress in (P.), 309
Digestive Organs, Progress in Diseases of the (P.), 11, 23, 46
Diphtheria (P.), 98 274, 275, 433
Disinfection (C.), 447
Disinfection of Rooms, The (C.), 5
Displacement of Tendons (C.), 272
Divided Sanitary Responsibility, 17
Doctors and Municipal Socialism, 410
Douches, The Composition of (C.), 167
Dowsing Radiant Heat Baths (W.), 212
Drainage Tubes, A Warning Concerning (C.), 115
Draining the Peritoneal Cavity, The Natural Method of (P.), 186
Drill in Schools, 1
Drug, A Popular (C.), 449
Drug Rashes (P.), 452
Dublin, City of, Hospital Nurses' Home (W.), 421
Duodenal Ulcer (P.), 24
Duodenum, Carcinoma of (P.), 24
Dysentery (P.), 47
Dysentery, Acute (P.), 209
Dysmenorrhœa, Ovarian, Spasmodic (C.), 133
Eclampsia, Cæsarian Section for (P.), 292
Eclampsia, The Treatment of (C.), 7
Eczema (P.), 310
Edelmann-Galton Whistle (P.), 168
Editor's Letter Box, 15, 141, 198, 230, 247, 265, 281, 369, 388, 423, 457
Education—Past and Future, 143
Education Bill, A Neglected Aspect of the, 261
Educational Problem, An, 320
Electrical Shocks, Accidental (C.), 5
Electrical Treatment of Abdominal Viscera (C), 358
Electro-plater's Dermatitis (P.), 310
Elementary Teaching: an Opening for Educated Women, 365
Elephantiasis of Exterior Genital Organs (P), 450
Embolism of the Superior Mesenteric Artery (P.), 187
Endocarditis, Malignant (C.), 45
Enteric Fever in the Army, The Persistence of (A.), 3

Enterectomy contrasted with Artificial Anus (O.), 342
Epilepsy (P.), 224
Epilepsy and Crime (O.), 358
Epilepsy, Death in (P.), 378
Epithelioma of the Skin (P.), 328, 397
Erysipelas and Puerperal Fever (O.), 205
Ether v. Chloroform (O.), 185
Evelina Hospital (W.), 123
Experiments on Animals (A.), 145
Exploiting a Prescription (A.), 59
Extraneous Organisms in Vaccine Lymph (O.), 204
Fasting (A.), 201
Fatigue as a Cause of Consumption (O.), 21
Feeble-minded, What to do with the (A.), 181
Fevers, Progress in (P.), 24, 47, 66, 380, 395
Fibroids, Supra-vaginal Hysterectomy for (P.), 433
Finger Prints and the Law (A.), 427
Fire and the Architects (A.), 181
Fires in Hospitals (A.), 269
"Flatites." The (A.), 251
Forceps, Use and Abuse of the (P.), 291
Fractures and Dislocations (P.), 48, 118
Friedenheim Hospital (W.), 158
Friedreich's Ataxy (P.), 151
Gall-stones (P.), 24
Gall-stones, Treatment of (O.), 22, 132
Gasserian Ganglion, Removal of the (P.), 452
Gastric Hæmorrhage (P.), 169
Gastric Physiology (P.), 23
Gastric Ulcer (P.), 169
Gastritis, Phlegmonous (P.), 11
General Medical Council, The, 162
Genito-Urinary Surgery, Progress in (P.), 99, 116, 133, 328, 347, 434, 450
"Give Him Air; He'll Straight be Well." 354
Glances at the Hospitals (W.), 106, 280, 298, 422, 440
Glasses, The Misuse of (O.), 394
Gold Miners' Phthisis (O.), 205
Gonorrhœa, The Treatment of (P.), 450
Gordon Hospital (W.), 73
Gout, The Treatment of (O.), 6
Gynæcology, Progress in (P.), 419, 432
"Habits," 57
Hæmorrhoids, Internal, Treatment of (P.), 451
"Half-Timers" (O.), 429
Hallux Valgus or Bunion (O.) 43
Hampstead Hospital Festival Dinner (W.) 176
Health Resorts (W.), 211, 316, 367, 386
Heart Disease, Exercise in, 234
Heart Disease, Rest in (O.), 377
Heavy Fees and Wealthy Patients (A.), 355
Hepatic Abscess (P.), 277
Heterogenesis, 283
Hip, Congenital Displacement of the (P.), 361
His Majesty and the Hospitals (W.), 141
Holidays, The, 128
Honourable Order of Spinsters, The, 313
Hospital Dispute at Torquay (W.). 30
Hospital Expenditure (W.) 366, 423
Hospital Festivals, Meetings, etc. (W.), 12, 29, 51 69, 315
Hospital Floors (W.), 265, 281, 300
Hospital and Home for Incurable Children (W.), 123
Hospital Reform in France (W.), 29
Hospital of St. Francis (W.), 296
Hospital for Sick Children (W.), 175
Hospital Sunday (W.), 191
Hospital Sunday in London, A Record (A.), 321
Housewifery Teaching in Belgium, 333
Housing of the Working Classes (O.), 222
Hydatid Disease of the Gall Bladder (P.), 291
Hydrocele, Bilocular, Intrapelvic and Scrotal (P.), 117
Hydrocele, Treatment of (P.), 435
Hydrophobia in South Wales (O.), 186
Hyperemesis Gravidarum (P.), 291
Hypnotism (O.), 148
Ice Charities (A.), 303
Idiosyncrasy, 216
Ills to which Flesh is not Heir, 121
Imperial Vaccination League, 371
Inaccuracy of Language (O.), 184
Inconsistencies, Our, 330
Inebriate Women (A.), 111
Infant Feeding (O.), 324
Infantile Paralysis, Spastic, Surgical Treatment of (O.), 414
Infantile Paralysis, The Treatment of (O.), 22
Infants at School (O.), 7
Infected Area, An (A.), 339
Infectious Diseases, The Law Relating to (W.), 278, 314, 348, 384, 438, 456
Insane, The Story of the, from Year to Year (W.), 31, 105, 246, 264, 280, 297, 351, 368, 387, 441
Insanity, The Influence of Acute Diseases upon (O.), 46
Insanity of Puberty and Adolesence (P.), 152, 153, 207
Insomnia, Treatment of, by Drugs (O.), 183
Intestine, Small, Extensive Resection of the (P.), 241
Intestines, Progress in Surgery of the (P.), 240
Isolation in Scarlet Fever (O.), 7
Jaundice, Epidemic (P.), 24
Jaundice, Obstructive (O.), 290
Jejunostomy (P.). 242
Kidney, A New Way of fixing the (O.), 239
Kidney, Floating (P.), 258
Kidney, Movable (P.), 328
King Edward's Hospital Fund (W.), 139
King's Chocolate, The (W.), 213
King's Dinner, The, 138
King's Hospital Fund, The (W.), 139
King's Illness, The, 233
King's Thank-offering, The (A.), 339
Knee-jerks (P.), 344
Labour—When does Labour Begin? (O.) 393
Labour, Obstructed, Treatment of (O.), 288
Lace Making as an Occupation, 331
Law, The, and Unqualified Practice (A.), 163
Lead Poisoning (O.), 325
League of Mercy at Marlborough House, The (W.), 174
Leprosy (O.), 166, 220
Lesson in Humility, A (A.), 145
Leucocytosis in Appendicitis (O.), 96
Leukoplakia (P.), 328
Light and X-Ray Treatment (P.), 327
Linen Thread, Surgical Uses of (O.), 150
Liver and Gall Bladder, Surgery of the (P.), 277 290
Liver, Wounds of the, (P.), 277
Liverpool Sanatorium for Consumptives (W.), 439
London Hospital, The (W.), 197, 421
London Hospital New Operating Department (W.), 156
London Hospitals, The (W.), 210
London Inquests (A.), 411
London Temperance Hospital (W.), 13
Lower Extremity (Surgery) (P.). 136
Lumbago and its Counterfeits (O.), 203
Lunacy Law Reform (A.), 145
Lunacy, The Prevalence of, in England and Wales (O.), 431
Lunacy in Scotland (W.), 385
Lunacy Statistics (O.), 167
Lupus (P.), 327, 397
Malaria in England (O.), 132
Malaria in the Roman Campagna (A.), 269
Manchester Royal Infirmary (W.), 332
Mastoid Suppuration, Operative Interference in (P.), 223
Medical Advertising (A.), 19
Medical Aspects of the West Indian Islands (O.), 147
Medical Curriculum, The, 401
Medical Etiquette (A.), 129
Medical Politics at the Antipodes (A.), 235
Medical Qualifications (A.), 35
Medical Schools, The, 402
Medical, Surgical and Hygienic Exhibition, 159
Medicine, Surgery and Gynæcology (A.), 269
Melancholia, Prognosis and Sequelæ of (P.), 25
Melancholia, Treatment of, (P.), 8
Meningitis (P.), 398
Mental Cases, Early (O), 289
Mental Changes in Visceral Disease (P.), 224
Mental Disorders, Relation of, to Bodily Disease (P.) 378
Mental Faculties, Localisation of the (O.), 63
Methylated Spirit Drinking (O.), 394
Metropolitan Hospital, The (W.), 71
Metropolitan Hospital Sunday Fund (W.), 68, 333
Micro-Organisms and Granulation Tissue (P.), 99
Micro-Organisms in Milk (P.), 209
Middle-Ear Disease, The Treatment of Chronic Non-Suppurative (P.), 168
Middlesex Hospital (W.), 195
Midwives Bill, The (A.), 111, 179; (W.), 231, 249
Midwives of the Future, The, 284
Milk Diphtheria (P.), 275
Miller Hospital, Greenwich (W.), 104
Mitral Stenosis, Surgery in (O.), 6
Modern Lettre de Cachet, A, 127
Modern Sociology, 27, 103, 121, 138, 155, 261, 295, 313, 331, 365, 383, 420, 437, 455
Molluscum Contagiosum (P.), 418
Morphia Poisoning, Salt Infusion in (O.), 115
Mosquitos and Sea-borne Malaria (O.), 289
Motor Speed, The Physiological Limits of, 443
Motoring for Health, 2
Mount Vernon Hospital Country Branch (W.), 122
Munich Royal School for Midwives (W.), 227
Muscular Tonus and the Reflexes (P.), 118
Mystery of Life, The (A.), 445
Nævi, Treatment of, by Electricity (O.), 131
Naso-Pharyngeal Lesions in Childhood (O.), 413
National Hospital for the Paralysed (W.), 12, 196
National Sanitary Organisation (A.), 33
Nephrotomy and Nephro-lithotomy (P.), 347
Nerve Deafness, A Case of Acquired (O.), 165
Nervous System, Prognosis of Diseases of the (W.), 171
Nephritis, Etiology of Chronic, (A.), 338
Neurology, Progress in (P.), 117, 150, 170, 206, 224, 344, 378, 397, 432
New Appliances and Things Medical, 11, 49, 67, 101, 137, 171, 189, 209, 225, 243, 277, 293, 311, 329, 347, 363, 398, 435, 453
New Milk (A.), 93
Nice Place to Live in, A (A.). 427
Nicotine, Action of, on Nerve Cells (P.), 118
"No Flies," 337
Normal Saline, Therapeutic Uses of (O.), 341
North-Eastern Hospital for Children (W.), 122, 141, 157
North-West London Hospital (W.). 30
Notes and News, 4, 20, 36, 60, 94, 112, 130, 146, 164, 182, 202, 218, 236, 250, 270, 286, 304, 322, 340, 356, 374, 392, 412, 428, 446
Obstetrics, Progress in (P.), 291
Official Visits to Private Patients (A.), 427
Omentofixation for Hepatic Cirrhosis (P.), 291
Ophthalmology, Progress in (P.), 66, 82, 100, 326
Organicidia Gastrica (O.), 449
Orthopædic Surgery, Progress in (P.), 360
Otitic Brain Abscess (P.), 224
Otitic Lateral Sinus Thrombosis (P.), 224
Otology (P.), 168, 187, 223
Otorrhœa and its Treatment (P.), 187
Our Child Population (A.), 411
Paddington Green Children's Hospital (W.), 104
Pancreas and Spleen, Progress in Surgery of the (P.), 259
Pancreatic Cysts (P.), 259
Paralysis, Family Periodic (P.), 152
Paralytic Deformities, Treatment of (P.), 360
Paraplegia, Syphilitic Spasmodic (P.), 398
Paratyphoid Infections (O.), 204
Patent Foods, 250
Peculiar People and the Law, The, 34
Pelvic Inflammation (P.), 433
Perineum, Rupture of the (O.), 395
Peritoneum, Surgery of the (P.), 186
Phthisis, Industrial (O.), 393
Phthisis, Intra-tracheal Injections in (A.), 251
Phthisis, Temperature in, 110
Physical Training and Free Meals (A.), 373
Physicians, Surgeons and Appendicitis (A.), 251
Pineapple Juice in Medicine (O.), 273
Placenta Prævia (O.), 430
Plague (P.), 47, 380
Plague as a Soil Infection (O.), 359
Pneumococcal Infection of the Peritoneum (P.), 186
Poplar Hospital for Accidents (W.), 14
Porous Walls (A.), 321
Posterior Drainage in Gunshot Wounds of the Abdomen (O.), 343
Pott's Disease, Extension in (O.), 415
Poverty, Hunger, and Dirt, 455
Practical Departments, 16, 23), 300, 369
Pregnancy after Double Ovariotomy (O.), 205
Pregnancy complicated by Epithelioma (P.), 292
Prophylactic Inoculation, 409
Prostate, Enlarged (O.), 21; (P.), 116, 434
Prostatic Enlargement, The Choice of Operation for (O.), 115
Psychiatry, Progress in (P.), 8, 25, 152, 207
Punctuality in Infant Feeding, 144
Pyrexia and Open Air (O.), 220
Pyrexia in the Puerperium (O.), 96
Queen of Holland, The (A.), 129
Rabies (P.), 418
Race and Physical Education, 215
Red Tape in Army Medical Affairs (A.), 285
Reflexes (P.), 344
Renal Calculus (P.), 99
Renal Diseases, Progress in (P.), 257
Revaccination, Efficacy of (A.), 321
Revaccination, Universal (O.), 343
Ridiculous Minister, A (A.), 303
Ringworm (P.), 452
Rodent Ulcer (P.), 328, 397
Roentgen Rays, The late Effects of the (A.), 339
Royal Army Medical Corps, The, 110
Royal Hospital of the Innocents (W.), 349
Royal Orthopædic Hospital (W.), 52, 107
Sacculus, Vesical (P.), 100
Saddle Nose, Subcutaneous Injection of Paraffin in (O.), 97
St. George's Hospital (W.), 69
St. Mary's Hospital Extension (W.), 55
St. Peter's Hospital (W.), 15
Samaritan Free Hospital (W.), 73
Sanatoria for the Consumptive Poor (A.), 391
Sarcoma (P.), 396
Scarlet Fever (P.), 395
School Nurses' Society, The, 267
Science and the Horse Dealer (A.), 373
Scientific Education, 426
Security of Tenure of Office for Sanitary Officials (A.), 181
Sham Nursing Homes (W.), 173
Shaving, The Sanitary Advantages of (A.), 391
Shortened Limbs (P.), 361
Sinuses, Callous (P.), 397
Skin Diseases, Progress in (P.), 310, 327, 451
Small-pox (P.), 9, 24
Small-pox and Country Holidays (A.), 93
Small-pox and Vaccinia, Concurrent (A.), 129
Soil and Seed (O.), 165
Soldiers and their Feet (A.), 285
South African Typhoid (A.), 235
Spa Physician, The, 92
Special Schools under the London School Board, 27, 103, 155
Spectacles for Iridemia (P.), 292
Spina Bifida (O.), 414
Splashing Sounds in the Stomach (P.), 11
Spleen, Movable (P.), 259

State Medicine, Progress in (P.), 9, 134, 222, 345, 361
Statistics, An Error in the Use of (O.), 431
Steam Organ, The (A.), 217
Stomach, Dilatation of the (O.), 323
Stomach, Progress in the Surgery of the (P.), 169
Streptococci (P.), 208
Strychnine, Overdosing with (A.), 129
Student of Medicine, The, 16, 32, 74, 108, 125, 142, 160, 178, 198, 213, 231, 248, 266, 282, 300, 318, 336, 352, 370, 388, 408, 424, 442, 457
Study of Medicine, The, 389
Summer Diarrhœa (C.), 357
Surgery of the Brain and Nerves (P.), 452
Surgery, General (P.), 48, 118, 133
Surgery, Genito-Urinary (P.), 99, 116, 133, 328, 347, 434, 450
Surgery of the Intestines (P.), 240
Surgery of the Liver and Gall Bladder (P.), 277
Surgery, Orthopædic (P.), 360
Surgery of the Pancreas and Spleen (P.), 259
Surgery of the Peritoneum (P.), 186
Surgery, Progress in (P.), 48, 168, 187, 224
Surgery of the Stomach (P.), 169
Surgery of the Vermiform Appendix (P.), 240
Symphisiotomy (P.), 291
Syphilis of the Stomach (P.), 23
Syphilis, Treatment of (P.), 133
Tabes (P.), 206, 344
Tabes due to Congenital Syphilis (P.), 170
Tabes Dorsalis, Anomalous Cases of (O.), 341
Tabes Dorsalis, Laryngeal Crises in (P.), 433
Tapping, Diagnostic, of the Chest (O.), 185
Temperance and Education, 444
Tenotomy in Fractures (O.), 150
Tenure of Hospital Appointments (A.), 19
Testis, Imperfectly Descended (P.), 435
Thrombi, Ante-mortem (P.), 99
Thrombosis of the Mesenteric Vessels (P.), 187
Thyrotomy (O.), 63
Tonsillotomy (O.), 184
Torbay Hospital, A Climax at (W.), 70
Town and Village Typhoid (O.), 377
Trade Unionism in High Places, 180
Transperitoneal Ligature of the Internal Iliac Artery (O.), 359
Trypanosoma (C.), 239
Tuberculosis (P.), 134
Tuberculosis in Asylums (A.), 285
Tuberculosis, Primary, of the Cervix, simulating Carcinoma (O.), 45
Tuberculosis, The Prevention of (A.), 93
Tuberculous Peritonitis, Treatment of (O.), 273
Tumour of the Corpus Callosum (P.), 151
Tumour of the Superior Vermis of the Cerebellum (P.), 151
Tumour, Sub-cortical, of Broca's Convolution (P.), 151
Typhoid Fever (P.), 188
Typhoid Fever, Diet in (O.), 273
Typhoidette (O.), 22
Ulcer, Rodent (P.), 328, 397
Unilateral Exclusion in Fæcal Fistula (P.), 241
Urethral Discharge, Chronic (P.), 450
Urinary Fever (P.), 116
Urology (P.), 257
Uterus, Retroflexion of the (O.), 183, (P.) 419
Vaccinate, Where to (O.), 98
Vaccination, Compulsory, An Example of (O.), 97
Vagina, Primary Sarcoma of the (P.), 433
Variola and Varicella (C.), 254
Ventilation (P.), 345
Victims of the War, 161
Victoria Hospital for Children, Chelsea (W.), 157
Victoria Hospital, Folkestone (W.) 51, 262
Virchow, Professor, Death of (A.), 411
Vision, Some Functional Disorders of (P.), 327
Voice Strain in Teachers (O.), 95
Volkmann's Contracture (P.), 225
Weaker Vessel, The (A.), 59
Weather and Mortality, 353
West London Hospital (W.), 72
Widal's Reaction (P.), 188
Whooping Cough, Paralysis in (P.), 378
Whooping Cough Statistics (O.), 287
Word Blindness (P.), 170, 397
X-ray Therapeutics, Progress in (P.), 379, 393
X-ray Treatment (P.), 327
Yellow Fever (P.), 396

### THE SPECIAL SECTION FOR NURSES.

Action by a Nurse against a Newspaper, 125
All He Had, 305
Amenities of Male Nurses' Co-operations, The, 253
Annual Conference of the Matrons' Council, 139
Annual Meeting of the Colonial Nursing Association, 205
Appointments, 9, 20, 45, 59, 67, 86, 101, 113, 124, 141, 169, 178, 191, 199, 217, 230, 244, 256, 271, 283, 295, 309, 325, 339, 351
Army Nursing Service Reserve and the South African War, The, 266
Bank Holiday, A, 251
Beyond the Seas, 35, 53
Beyond the Seas: the Lunatic Asylum, Jamaica, 82
Beyond the Seas: Nursing in India, 149
Beyond the Seas: Nursing in a Japanese Fever Hospital, 95
Beyond the Seas: Six Months in Tierra del Fuego, 322
Bombay Hospitals, Sketches from Life in, 348
Book and its Story, A, 46, 88, 127, 143, 179, 219, 258, 298, 340
Capetown to Salisbury, From, 40
"Case against Hospital Nurses, The," 18, 96
Character Sketch, A, 99
Children's Surgical Ward, The, 36
Cholera in Egypt, 319
Coronation Duty at Westminster Abbey, On, 269
Coronation Entertainment by the Nurses of the Royal Isle of Wight County Hospital, 269
Cypriot Babies, 304
Day with a District Nurse, A, 252
Death of a Crimean Nurse, 151
Death in our Ranks, 8, 45, 59, 113
"Distinction with a Difference," A, 81
District Maternity Nursing in London, 6
District Nurse and the "Pianner Lidy," The, 310
District Training under Queen Victoria's Jubilee Institute, 323
Drawing-room Meetings, 135
Dumb Ward Pets, 267
East End Mothers' Home, 103
Echoes from the Outside World, 11, 22, 47, 60, 75, 87, 102, 115, 128, 142, 156, 168, 180, 193, 206, 218, 232, 245, 257, 272, 285, 297, 312, 326, 341, 354
End of the Coronation, The, 281
Enemata, Different Kinds of, 98
English Nursing in Rome, 153
English Sisters and Boer Leaders, 240
Everybody's Opinion, 10, 21, 43, 56, 73, 85, 100, 114, 126, 140, 155, 167, 178, 190, 204, 216, 229, 242, 254, 270, 284, 295, 310, 323, 338, 353
Examination Questions:—
  Rheumatic Fever, How to Manage a Severe Case of, 203
  Sponging in Typhoid Fever, 33
  Support of Helpless Patients in Bed, The, 152
Female Nurses for Male Lunatics, 8
Fête in Aid of St. George's Hospital, 201
Fever Hospital and House of Recovery, Cork Street, Dublin, 199
Fever in the Milkcan, 280
Garden Party at Gartlock Asylum, 177
Home made Hospital Preserves, 349
Holidays, Some Ideas about, 337
Hospital Ship for Fishermen, A, 153
Interesting Experience in the Philippines, 202
Lectures on Gynæcology to Nurses, 16, 80, 108, 134, 162
Lectures to Nurses on Anatomy, 5, 28, 66, 94, 120, 148, 174, 224, 250, 303
Lectures to Nurses on Medical Nursing, 186, 212, 238, 261, 318, 346
London Hospital Pension Scheme for Nurses, The, 187
Male Nurses in General Hospitals, 20
Matrons in the Burgher Concentration Camps, 121
Metropolitan Nursing Association, The, 72
My Masseuse, 56
Native Indian Midwives, 124
Naval Review, The, 282
Not a Typical Patient, 41
Notes on News from the Nursing World, 1, 13, 25, 49, 63, 77, 91, 105, 117, 131, 145, 159, 171, 183, 195, 209, 221, 235, 247, 261, 275, 287, 301, 315, 330, 343
Notes and Queries, 12, 23, 48, 61, 76, 89, 104, 116, 129, 144, 157, 170, 181, 194, 207, 220, 233, 246, 259, 273, 286, 299, 313, 327, 342, 355
Novelties for Nurses, 19, 42, 59, 84, 101, 125, 152, 244, 320
Nurse of the Future, The, 293
Nurse's Action at Barnstaple, A, 349
Nurse's Life in India, A, 137
Nurses' Bookshelf, The, 141, 192, 243, 255, 283
Nurses of Friedenheim Hospital, The, 154
Nurses of Guy's Hospital, The, 30
Nurses' Hostel: Opening of the New Block, The, 166
Nurses of the London Homœopathic Hospital, 164
Nurses of University College Hospital, The, 239
Nurses of Wandsworth and Clapham Union Infirmary, The, 225
Nursing in a Bible Mission Van, 291
Nursing in the Burgher Camp, Kimberley, 56
Nursing in the Burgher Concentration Camps, 111
Nursing in Concentration Camps in Krugersdorp, 151
Nursing Diphtheria in a Girls' Home, 228
Nursing of the Insane, The, 336
Nursing in an Italian Hospital, 83
Nursing Private Patients in Egypt, 306
Nursing, Private, in North Germany, 347
Nursing, Private, in South Africa, 17
Nursing in Rome, English, 153
Nursing at the Royal Portsmouth Hospital, 333
Nursing and Social Life, 309
Off-duty Hours, For, 215
Opening of the Henriette Raphael Nurses' Home at Guy's Hospital, 200
Openings for Nurses, 29
Outdoor Uniform, 214
Out-patients in the Transvaal, 188
Papers for Private Nurses, 38, 54, 84
Plaistow Maternity Charity and District Nurses' Home, 167
Plants Suitable for Hospital Decoration, and How to Tend Them, 177
Position of a Private Nurse, The, 175
Presentations, 35, 53, 74, 84, 101, 112, 125, 154, 169, 191, 217, 228, 240, 256, 271, 311, 321, 335
Quarantine Station of El Tor, The, 241
Queen at the Alexandra Hospital, The, 9
Queen Alexandra's Imperial Nursing Service, 4
Queen Alexandra's Naval Nursing Service, 109
Queen and the Imperial Yeomanry Hospitals, The, 265
Queen Victoria's Jubilee Institute for Nurses, 187
Queen's Visit to the Louise Margaret Hospital, Aldershot, The, 163
Reading to the Sick, For, 10, 23, 45, 61, 76, 89, 103, 116, 129, 144, 157, 169, 181, 192, 207, 220, 231, 246, 256, 273, 286, 296, 313, 325, 339, 350
Red Cross Nurses of Germany, The, 268
Retiring Matron of St. Thomas's Hospital, The, 227
Royal British Nurses' Association, 70, 165
Royal British Nurses' Association and the Midwives Bill, 112
Royal National Pension Fund for Nurses, 68
"Silent Hour" in a Children's Ward, The, 321
"Silent Hour" in Hospital Wards, A, 294
Suicide of an Assistant Superintendent Nurse at Crumpsall Workhouse Infirmary, 320
Three Months' Midwifery Training, 278
Travel Notes, 24, 62, 90, 103, 113, 130, 153, 182, 208, 231, 234, 244, 260, 273, 300, 314, 328, 352, 356
Visit to the Bergen Leper Hospital, 213
Visit to a Deaconesses' Hospital in the Black Forest, A, 19
Visit to the Foundling Hospital at Florence, A, 7
Visit to the Hospital for Incurables at Naples, A, 308
Visit to an Indian Leper Home, A, 292
Visit to the Leper Hospital, Pretoria, A, 32
Visiting Native Patients in India, 34
Wanted, A Probationer: also a Cook, 55
Week in a Nurses' Holiday Home, A, 334
Workhouse Infirmary Nursing Reform, 123, 136

Printed by SPOTTISWOODE & CO. LTD., New Street Square, E.C.; and Published by the Proprietors, THE SCIENTIFIC PRESS, LIMITED, at 28 & 29 Southampton Street, Strand, London, W.C.

### NOTICE TO CORRESPONDENTS.

All MSS., Letters, Books for Review, and other matters intended for the Editor should be addressed The Editor "The Hospital," The Hospital Building, 28 & 29 Southampton Street, Strand, W.C.

The Editor cannot undertake to return rejected MS., even when accompanied by stamped directed envelope.

**Notice to Advertisers and Subscribers.**

All Advertisements, Orders for Copies of The Hospital and Business Communications should be addressed to **THE MANAGER, (Not to the Editor)**, The Hospital, 28 & 29 Southampton Street, Strand, London, W.C.

The Cost of Subscription, post free, is as follows.—Per week, 2½d.; per quarter, 2s. 8d.; per half-year, 5s. 3d.; per annum, 10s. 6d. Foreign:—Per annum, 15s. 2d., payable, in all cases, in advance.

**NOTICE.—Vol. XXXI. of "The Hospital" (October 1901, to March 1902), in handsome cloth binding will shortly be on sale at the office of this Journal, price, 7s. 6d. Cases for binding the Numbers contained in this Volume 1s. 6d. Index to Vol. XXXI., 2½d., post free.**

**The Monthly Part for April is now ready. Price 6d., by post 8d.**

### PRACTICAL DEPARTMENTS.

### THE STUDENT OF MEDICINE.

### APPOINTMENTS.

**Harold Cotterell Adams**, L.R.C.P.Lond., M.R.C.S., appointed Medical Officer for the Paignton and Marldon Districts. **Hector Allan**, M.B., C.M.Aberd., appointed Medical Officer of Health to the Whalley Bridge Urban District Council. **Arthur Stanley Barnes**, M.D., M.R.C.P.Lond., B.Sc., appointed Senior House Physician to the National Hospital for the Paralysed and Epileptic. **Lancelot M. Breton**, M.R.C.S., L.R.C.P.Lond., appointed Medical Officer and Public Vaccinator to No. 3 District of the South Stonham Union. **Charles Cecil Bullmore**, L.R.C.P., L.R.C.S.Edin., L.F.P.S.Glasg., appointed Medical Officer for the Falmouth and Truro Port Sanitary Authority. **E. Farquhar Buzzard**, M.A., M.B., M.R.C.P.Lond., appointed Assistant Physician to the Belgrave Hospital for Children. **Helena G. Jones**, M.B.Lond., appointed Assistant Medical Officer to the District Asylum, Mullingar. **G. G. Phillips**, M.R.C.S., L.S.A., appointed Medical Officer of Health to the Tickhill Urban District Council.

### Medical Appointment.

CENTRAL LONDON SICK ASYLUM DISTRICT.

ELECTION OF ASSISTANT MEDICAL OFFICER.

The Managers require the services of a FIRST ASSISTANT MEDICAL OFFICER, for duty at either of their Asylums at Cleveland Street or Hendon, as may be required.

Salary £120 per annum, with Board and Residence.

Applicants must possess a qualification, both in medicine and in surgery, according to the regulations of the Local Government Board, and a good knowledge of dispensing.

The duties are: To give the Medical Superintendent such assistance as he may require, and to dispense such medicines as may be ordered.

The appointment will be made subject to the approval of the Local Government Board, and to the provisions of the Poor Law Officers' Superannuation Act, 1896, and will be, at first, for a period of twelve months, renewable at the termination of that period for a further period not exceeding two years in all, at the discretion of the Managers.

Applications, with copies only of not more than three recent testimonials, are to be forwarded to the undersigned.

By order of the Managers,

Clerk's Office, FRED. W. BAILEY, Clerk.
Cleveland Street Asylum, Cleveland Street, London, W.
April 2nd, 1902. (317)

**Tendencies to Consumption:**

**HOW TO COUNTERACT THEM.**

**By J. D. E. MORTIMER,**

M.B. Lond., M.R.C.S., L.S.A.,

Author of "Home Nursing of Sick Children."

Crown 8vo. cloth, **2/6.**

*To be obtained of all Booksellers, or of*

THE SCIENTIFIC PRESS, LTD., 28 & 29 Southampton Street, Strand, London, W.C.

